# Beyond M1/M2: The Pivotal Role of Macrophage Metabolic Reprogramming in Chronic Bone Disease and Targeted Intervention

**DOI:** 10.3390/ijms27093731

**Published:** 2026-04-22

**Authors:** Qiao Wan, Zeling Fang, Jiarong Shi, Yu Jiang, Hua Jin, Chuangwei Sui, Xupeng Liu, Fangyu An, Yanxia Zhang, Zhendong Chen, Fan Ding, Chunlu Yan

**Affiliations:** 1 School of Tradional Chinese and Werstern Medicine, Gansu University of Chinese Medicine, Lanzhou 730000, China; 2 School of Basic Medicine, Gansu University of Chinese Medicine, Lanzhou 730000, China; 3 Clinical College of Chinese Medicine, Gansu University of Chinese Medicine, Lanzhou 730000, China; lanzhoujinhua@126.com; 4 Teaching Experiment Training Center, Gansu University of Chinese Medicine, Lanzhou 730000, China; 5 Scientific Research and Experimental Center, Gansu University of Chinese Medicine, Lanzhou 730000, China; 6 The National Institute of Dunhuang Medicine, Gansu University of Chinese Medicine, Lanzhou 730000, China

**Keywords:** chronic bone diseases, macrophage polarization, immunometabolism, metabolic reprogramming, bone homeostasis

## Abstract

The progression of chronic bone diseases is intricately linked to dysregulated macrophage polarisation. However, a comprehensive understanding of the complex interplay between macrophage polarisation and metabolic reprogramming in the context of bone disorders remains elusive. Thus, this review conducted a systematic search of major databases, including PubMed, using combinations of keywords such as “macrophage polarisation,” “immunometabolism,” “metabolic reprogramming,” and “chronic bone diseases” (including “osteoporosis,” “osteoarthritis,” and “periodontitis”). Inclusion criteria prioritised original research published within the last five years to capture recent advances. Diverging from previous reviews constrained by the classical M1/M2 dichotomy, this article aims to delineate the heterogeneity and functional plasticity of macrophages within the bone microenvironment, emphasising metabolic reprogramming as a central mechanism driving the dynamic behaviour of macrophages across various skeletal pathologies. Furthermore, this review highlights the pivotal roles of specific metabolites—such as succinate, itaconate, and citrate—within the osseous microenvironment, underscoring their influence on macrophage phenotypic transitions and the regulation of bone metabolic homeostasis. Finally, this article envisages innovative therapeutic strategies targeting the “metabolism–immunity axis,” encompassing the design of nano-delivery systems to modulate macrophage metabolism, the utilisation of engineered extracellular vesicles, the development of immunometabolism-modulating biomaterials, and the exploration of naturally occurring bioactive molecules. Based on these findings, the present work proposes the “metabolism–immunity–skeleton” axis as a theoretical framework, thereby establishing a robust foundation for the development of precision metabolic immunotherapy tailored to a spectrum of chronic bone diseases.

## 1. Introduction

Chronic bone diseases, including osteoporosis, rheumatoid arthritis, and periodontitis, represent an escalating global health burden characterized by the disruption of skeletal homeostasis and persistent inflammation [1,2,3]. The maintenance of bone integrity relies on the precise coordination between bone-resorbing osteoclasts and bone-forming osteoblasts, a process orchestrated by the immune system within the “osteoimmune” microenvironment [4]. Among the diverse immune cell populations, macrophages have emerged as the central conductors of this interplay. Traditionally, macrophage function in bone has been viewed through the lens of a dichotomous M1 (pro-inflammatory/osteoclastogenic) and M2 (anti-inflammatory/osteogenic) polarization model [5]. While this paradigm has provided a fundamental framework for understanding pathological bone loss—where a skew towards M1 phenotypes drives inflammation and tissue destruction—it increasingly fails to capture the full complexity of macrophage behavior observed in vivo.

What are the fundamental drivers of functional differentiation of macrophages? The emerging field of “immunometabolism” provides a key perspective. Cellular metabolism is not only a passive process that provides energy for function but also a source of signals that actively determine cell fate and function. It has been shown that M1 polarisation relies heavily on rapid energy supply from glycolysis and pentose phosphate pathways, whereas M2 polarisation favours oxidative phosphorylation and fatty acid oxidation to support long-term function [6]. This metabolic reprogramming is not limited to glucose and lipids but extends to the profound remodelling of amino acid metabolism and mitochondrial function. More importantly, the intermediates produced in metabolic pathways can themselves serve as important signalling molecules that directly guide macrophage polarisation and functional outputs by modulating key transcription factor activities, epigenetic modifications, and inflammatory vesicle activation. Therefore, in chronic bone disease, the local abnormal metabolic microenvironment (e.g., high glucose, lipid accumulation, and hypoxia) is likely to be the core upstream mechanism leading to an imbalance in macrophage polarisation, which in turn drives the vicious circle of “chronic inflammation-bone metabolism disorders” [7].

Based on this knowledge, targeting the ‘metabolic-immune’ axis has become a promising new strategy to intervene in chronic bone diseases. These include the use of nano-delivery systems to precisely regulate the metabolic pathways of macrophages in the lesion, the design of biomaterials with immunomodulatory functions to guide macrophages towards a reparative phenotype through their physicochemical properties or the release of specific ions [8], and the development of extracellular vesicles derived from mesenchymal stem cells (MSCs) or macrophages themselves as natural therapeutic vectors. These strategies aim to remodel the bone immune microenvironment at the source and transform destructive inflammation in pathological states into favourable conditions that promote regenerative repair [9]. Despite significant progress, this field still faces many challenges. For example, macrophage metabolism-phenotype associations in complex in vivo microenvironments are highly dynamic and context-dependent, and the dominant metabolic abnormalities and key macrophage subpopulations may differ between chronic bone diseases or at different stages of the same disease. Consequently, efficient and specific delivery of metabolic interventions to bone foci with spatially and temporally controllable modulation is still not an easy task. Therefore, there is an urgent need to integrate multidisciplinary technologies, such as metabolomics, single-cell spatial transcriptomics, and in vivo imaging, to systematically map the metabolic immunity of macrophages in bone tissues under physiological and pathological conditions, and to elucidate the specific molecular mechanisms by which they regulate bone homeostasis [10].

This review proposes the ‘metabolic-immune-bone’ axis as a theoretical framework, aiming to systematically elucidate the central role of macrophage dynamics driven by metabolic reprogramming in chronic bone diseases. We first analyse macrophage heterogeneity beyond M1/M2, followed by an in-depth discussion on how dysregulated energy metabolism serves as a common mechanism in chronic bone disease, with a focus on how sugar, lipids, amino acids, and mitochondrial metabolic reprogramming specifically regulate macrophage fate. We then review innovative therapeutic strategies targeting this key axis, including nanotechnology, biomaterials, extracellular vesicles, and naturally active molecules. Finally, we envision future research directions and challenges for clinical translation with the aim of providing a theoretical rationale and novel perspectives for the development of precision bone disease therapies based on immunometabolic modulation.

## 2. Macrophages in the Bone Microenvironment

### 2.1. M1/M2 Dynamic Balance: The Key to Pathological Regression of Chronic Bone Disease

Macrophage polarisation and its regulatory role in the bone immune microenvironment have become central to understanding the pathogenesis of chronic bone disease [11]. Macrophages are highly plastic and can polarise into the M1 and M2 types with very different functions when stimulated by different microenvironmental signals, and the dynamic balance between the two precisely regulates bone metabolism, whereas their imbalance is a key factor driving the development of multiple chronic bone diseases [12]. In a study by Feng et al. [13], in vitro experiments have revealed that, in ankylosing spondylitis, the highly expressed chemokine C-X3-C motif chemokine ligand 1 exacerbates inflammation and bone destruction by activating the nuclear factor kappa B (NF-κB) signalling pathway, driving macrophage polarisation towards the pro-inflammatory M1 phenotype, and promoting osteoclast differentiation. Therefore, M1-polarized macrophages have been shown to be a central link in driving pro-inflammatory bone destruction in ankylosing spondylitis. In contrast to the bone-destructive nature of M1 macrophages, M2-polarized macrophages actively contribute to bone protection and regeneration. In a study by Nakao et al. [14], in vitro experiments revealed that exosomes secreted by gingival tissue-derived MSCs (GMSCs) were able to convert the macrophage phenotype from pro-inflammatory M1 to anti-inflammatory M2 under tumour necrosis factor-α (TNF-α) stimulation. In a mouse model of periodontitis, GMSC-derived exosomes significantly inhibited bone loss [14]. This phenomenon was attributed to the infiltration of M2 macrophages and their anti-inflammatory effects, suggesting their potential for treating inflammatory bone immune diseases. Furthermore, Qin et al. [15] demonstrated that bilobalide is able to dose-dependently promote macrophage polarisation towards the M2 phenotype, as evidenced by significant up-regulation of the M2-type activation markers CD206 and CD163. Mechanistically, by up-regulating the expression of sirtuin 3, bilobalide not only inhibits osteoclast differentiation but also dominates the macrophage transition to the M2 phenotype [15]. When sirtuin 3 was knocked down using small interfering RNA, the ability of white fruit lactones to induce M2 polarisation was significantly attenuated [15], demonstrating that sirtuin 3 is a key upstream regulator of M2 polarisation. In vitro experiments further validated the osteoprotective role of M2 macrophages. In an ovariectomised (OVX) osteoporosis mouse model, not only were bone density and microarchitecture significantly improved in the *Leuconostoc*-treated group, but the number of CD206-positive M2 macrophages in the bone tissue was also significantly increased [15]. This suggests that drug-induced M2 polarisation is highly correlated with the osteoprotective effects in vivo. Therefore, M2-polarized macrophages are important for maintaining bone homeostasis and combating osteoporosis through their anti-inflammatory properties and potential bone-enhancing effects, inhibiting the over-activation of bone resorption while actively promoting bone repair and regeneration.

Notably, it has been found that the differentiation fate of macrophages goes beyond the classical M1/M2 polarisation and involves a critical choice to differentiate towards osteoclasts or pro-angiogenic subpopulations. Zheng et al. [16] demonstrated that this process is precisely regulated by Raf kinase inhibitory protein (RKIP). Their study revealed that RKIP inhibits the inactivation of CDC42 by competitively binding to Rho GTPase-activating protein, thereby positively regulating the differentiation of macrophages into osteoclasts and promoting bone resorption. Further, RKIP inhibits the pro-angiogenic gene expression driven by HIF-1α by promoting ubiquitinated degradation of HIF-1α in the nucleus [16]. This implies that RKIP simultaneously promotes bone resorption and inhibits H-type angiogenesis coupled with bone formation. Furthermore, in an ovariectomy-induced osteoporosis model, knockdown or inhibition of RKIP in macrophages prevented ovariectomy-induced bone loss by inhibiting bone resorption and promoting bone formation [16]. In vivo experiments further confirmed that the increase in bone mass due to RKIP deficiency was mediated by the inhibition of osteoclast differentiation and increased macrophage differentiation into pro-angiogenic subpopulations [16]. Therefore, targeting RKIP to regulate the direction of macrophage differentiation provides a novel therapeutic strategy for the treatment of chronic bone diseases, such as osteoporosis.

In conclusion, the dynamic balance between M1/M2 macrophages is crucial for maintaining the homeostasis of the body’s bone immune microenvironment, as well as for the pathological regression of chronic bone diseases. The dominance of M1 polarisation leads to a vicious cycle of inflammation and bone destruction, whereas tilting the macrophage differentiation fate towards M2 polarisation or altering the macrophage differentiation fate by modulating nodes, such as RKIP, in the direction of bone protection and repair, can help restore bone homeostasis. Targeted regulation of this homeostatic system provides a central theoretical basis and a potential therapeutic strategy for the treatment of chronic bone diseases such as ankylosing spondylitis, periodontitis, and osteoporosis. A deeper understanding of homeostatic dysregulation, as well as the exploration of finer subtypes and functional plasticity of macrophages, will provide new targets and strategies for the development of precise immunomodulatory therapies for chronic bone diseases [17].

### 2.2. Beyond M1/M2: New Insights into Macrophage Heterogeneity and Functional Plasticity in Chronic Bone Disease

**Figure 1:**
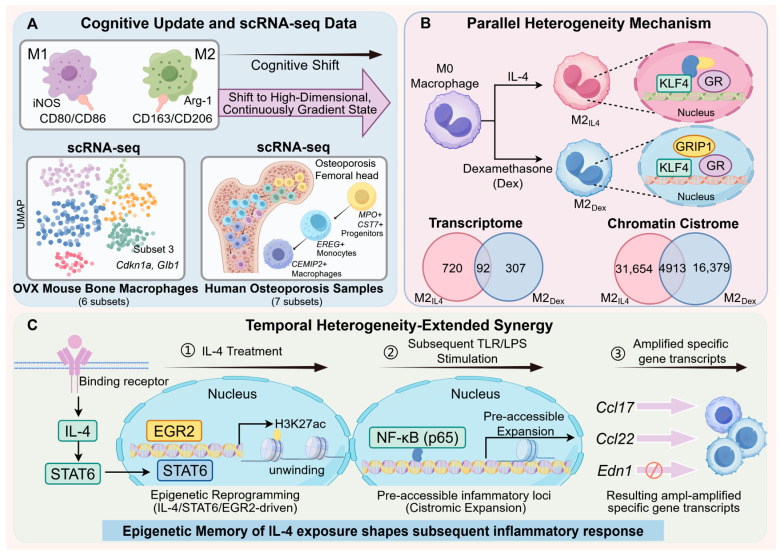
Heterogeneity and functional plasticity of macrophages in the bone immune microenvironment. (**A**) Evolution of the macrophage recognition paradigm. A shift from the traditional M1/M2 binary model towards a higher-dimensional continuous gradient of states. In vivo single-cell RNA sequencing (scRNA-seq) data revealed distinct subpopulations: bone macrophages in ovariectomised mice differentiate into six subpopulations, with Subset 3 characterised by ageing markers (*Cdkn1a*, *Glb1*); simultaneously, human osteoporotic samples span seven subpopulations, demonstrating pseudo-time trajectories of differentiation from *MPO+*/*CST7+* progenitor cells to mature *CEMIP2+* macrophages. (**B**) Parallel heterogeneity mechanisms. Although both exhibit an M2-like phenotype, interleukin-4 (IL-4) and dexamethasone (Dex) induce distinct epigenomic and transcriptomic profiles, respectively. The stimulus-specific integration of the co-factor GR-interacting protein 1 (GRIP1) with the transcription factors kruppel-like factor 4 (KLF4) and the glucocorticoid receptor (GR) underpins this divergence. This is evidenced by minimal overlap between the two in both the transcriptome (92 shared genes) and chromatin cis-regulatory elements (4913 shared regions). (**C**) Temporal heterogeneity driven by extended synergistic effects. Initial IL-4 exposure triggers signal transducer and activator of transcription 6 (STAT6)/early growth response 2 (EGR2)-mediated epigenetic reprogramming, which uncoils chromatin by increasing H3K27ac modification. Under subsequent Toll-like receptor (TLR)/lipopolysaccharide (LPS) stimulation, this epigenetic memory promotes cistromic expansion, enabling nuclear factor kappa B (NF-κB) (p65) to recruit to pre-opened inflammatory sites. Consequently, the transcription of specific genes (such as *Ccl17* and *Ccl22*) is significantly amplified, whilst other genes (such as *Edn1*) are suppressed, fully demonstrating stimulus-history-dependent functional plasticity. This figure was generated using Figdraw (www.figdraw.com), with the copyright code PYYAI062a2.

Traditionally, macrophages have been classified into pro-inflammatory M1 macrophages and anti-inflammatory/repair M2 macrophages. However, with the development of high-throughput technologies, such as single-cell RNA sequencing (scRNA-seq), researchers have found that macrophage heterogeneity goes far beyond this binary M1/M2 classification [18,19,20]. For example, bone macrophage gene profiles in OVX mice showed up-regulation of genes related to oxidative stress, cellular senescence, and apoptosis, and scRNA-seq analysis revealed that bone macrophages heterogeneously clustered into six subpopulations expressing proliferative, inflammatory, and anti-inflammatory traits [21] (Figure 1). This suggests the existence of more refined macrophage subtypes and functional states that may play specific roles in disease progression. A previous study by Deochand et al. [22] revealed that, even if they belong to the same M2-like phenotype, primary mouse bone marrow-derived macrophages induced by interleukin-4 (IL-4) and glucocorticoids exhibit significant heterogeneity (Figure 1). Transcriptome analyses revealed that IL-4-induced M2_IL4_ and dexamethasone-induced M2_Dex_ cells contained 720 and 307 differentially expressed genes, respectively, of which only 92 were shared [22]. At the epigenetic level, M2_IL4_ and M2_Dex_ have 31,654 and 16,379 chromatin-accessible differentially expressed regions, respectively, of which only 4913 are shared [22]. These data suggest that the activation state of macrophages is a complex spectrum and that the macrophage polarisation state mediated by different activation conditions differs significantly across the transcriptome and epigenome. Another study provided insights into the highly heterogeneous nature of macrophages in the femoral head tissue of patients with osteoporosis using scRNA-seq [23]. Researchers have identified seven different macrophage subpopulations, including granulocyte-monocyte progenitors with high expression of *MPO* and *CST7*, intermediate monocytes marked by *EREG* and *THBS1*, and macrophages marked by cell migration-inducing and hyaluronan-binding protein 2 (*CEMIP2*) [23] (Figure 1). The unique gene expression profiles shared by these sub-populations suggest that they are involved in various biological processes. Notably, these macrophages do not strictly conform to the traditional M1/M2 polarisation model but rather exhibit a continuous and diverse activation state [23]. These findings provide a cellular basis and a new perspective for understanding macrophage heterogeneity in the bone immune microenvironment.

Furthermore, under physiological and pathological conditions, the intrinsic molecular microenvironment of an organism is usually characterised by the simultaneous presence of multiple pathogen-associated molecules, cytokines, and metabolites, thus constituting an in vivo environment in which multiple signals coexist. This complex combination of signals can induce macrophages to form multiple unstable and transitional forms of polarisation rather than being limited to a simple M1/M2 dichotomy [24]. In addition, studies have overturned the conventional wisdom that IL-4 and Toll-like receptor (TLR) signalling are simply antagonistic, establishing ‘extended synergy’ as an important paradigm for macrophage functional plasticity [25] (Figure 1). These results confirmed that IL-4 pre-treatment radically alters macrophage responsiveness to TLR stimulation. This shift in the functional state is gene-specific; for example, it significantly enhances the responsiveness of genes, such as *Ccl17* and *Ccl22*, to lipopolysaccharide (LPS), while inhibiting a subset of genes [25]. This effect has been termed ‘extended synergy’, and its central mechanism is epigenetic reprogramming driven by the IL-4/signal transducer and activator of transcription 6 signalling pathway [25]. This phenomenon has been observed in murine and human macrophages of different origins (embryonic and mononuclear), suggesting an evolutionarily conserved regulatory mechanism [25]. This mechanism reveals the deeper logic of the macrophage response to complex microenvironmental signals, that is, macrophage functional plasticity and heterogeneity arise not only from different signals but also from the sequence and history of signal exposure. Thus, macrophage heterogeneity and functional plasticity may be jointly determined by (1) signal-specific transcriptional and epigenetic programming, (2) signal timing-dependent ‘extended synergistic effects’, (3) complex integration of multiple signals at the molecular level, and (4) the resulting functional subpopulations with unique gene modules. Understanding these specific molecular mechanisms is critical for the development of precise bone immunotherapy strategies that target specific macrophage subpopulations or modulate their functional plasticity.

In summary, macrophage heterogeneity and functional plasticity are central drivers of the pathophysiology of chronic bone disease [26,27]. A deeper understanding of macrophage subtypes beyond the M1/M2 classification and their dynamics in the bone microenvironment will provide new targets and strategies for the diagnosis and treatment of chronic bone diseases, such as osteoporosis and rheumatoid arthritis [11,28]. Future studies should utilise advanced technologies such as single-cell histology, multicellular histology, and spatial transcriptomics to further reveal the specific subtypes of macrophages in bone diseases and their molecular mechanisms to develop more precise immunomodulatory therapies.

## 3. Metabolic Reprogramming: Common Mechanisms and Cutting-Edge Perspectives in the Development of Chronic Bone Disease

### 3.1. Dysregulated Energy Sensing: From Energy Crisis to Bone Homeostatic Imbalance

Energy imbalance is closely associated with skeletal homeostasis, and energy sensors serve as key hubs through which cells perceive energy status and regulate bone metabolism [29,30]. AMP-activated protein kinase (AMPK) is an important regulatory kinase; as a cellular energy sensor, it is highly sensitive to increases in the AMP/ATP ratio and monitors cellular energy levels [31]. Recent research by Qin et al. [32] indicates that the glucagon-like peptide-1 receptor (GLP-1R) agonist semaglutide can restore AMPK phosphorylation levels by activating the GLP-1R-PKA-AMPK-PFKFB3 signalling axis, thereby upregulating the expression and activity of PFKFB3. This regulation inhibits the overactivation of glycolysis under inflammatory conditions, whilst significantly enhancing mitochondrial oxidative phosphorylation function [32]. Consequently, the ATP supply in chondrocytes shifts from an over-reliance on glycolysis to a balanced state where glycolysis and oxidative phosphorylation provide energy synergistically, ultimately restoring cellular energy homeostasis and promoting cartilage repair [32]. The study further demonstrated, using *Prkaa1* gene (encoding AMPK) knockout mouse models, that the chondroprotective effects of semaglutide were almost entirely lost following AMPK knockout [32]. Consequently, by sensing energy crises and reprogramming cellular metabolic pathways, AMPK acts as a key molecular switch linking energy imbalance to bone homeostasis imbalance; targeting the AMPK signalling axis is expected to become a new strategy for treating metabolic bone diseases. Similarly, Li et al. [33] found that, in a diabetic osteoporotic pathological environment, hyperglycaemic stimulation significantly downregulates the expression of the N6-methyladenosine reader protein (e.g., insulin-like growth factor 2 mRNA-binding protein 2 [IMP2]), thereby reducing the stability of *LKB1* mRNA and leading to the inhibition of the LKB1-AMPK signalling axis. Specifically, reduced AMPK phosphorylation levels decrease the expression of mitochondrial fusion proteins MFN1, MFN2 and OPA1, promoting mitochondrial fragmentation, loss of membrane potential and disruption of cristae structures; simultaneously, it downregulates the key fatty acid β-oxidation enzymes CPT1A and CPT2, impairing the cell’s ability to generate energy from fatty acids [33]. This energy metabolism dysfunction not only directly impairs the M2 polarisation of macrophages in bone tissue, but also indirectly inhibits the osteogenic differentiation capacity of bone marrow mesenchymal stem cells by reducing the secretion of anti-inflammatory factors transforming growth factor-β (TGF-β) and interleukin-10 (IL-10), ultimately exacerbating bone loss [33]. Conversely, overexpressing IMP2 or activating the LKB1-AMPK signalling axis in macrophages can restore mitochondrial homeostasis and fatty acid oxidation flux, providing M2 macrophages with sufficient ATP and metabolic substrates, thereby improving the imbalance between bone formation and resorption [33]. Consequently, targeting IMP2-mediated N6-methyladenosine modification and its downstream LKB1-AMPK energy-sensing network holds promise as a novel strategy for reversing diabetes-associated bone homeostasis imbalance. Furthermore, bone homeostasis depends on the dynamic balance between bone formation and resorption, and the high energy demand of osteoclasts, the main effector cells of bone resorption, makes energy metabolism a key link in the regulation of bone homeostasis. In vitro studies have shown that gonadotropins (FSH) promote osteoclast differentiation and bone resorption [34]. Correspondingly, FSH receptor-deficient osteoclasts exhibit reduced osteoclast activity and impaired energy metabolism in vitro, as evidenced by down-regulation of the osteoclast proteins CTSK and MMP9, reduced ATP levels, and decreased rates of glycolysis [34]. These data suggest that FSH meets osteoclast energy requirements through metabolic reprogramming; however, its over-activation may also trigger an imbalance in energy allocation, leading to hyper-resorption. Furthermore, FSH receptor deficiency down-regulates osteoclast energy metabolism through nicotinamide adenine dinucleotide (NAD^+^) depletion and malate-aspartate shuttle (MAS) dysregulation [34]. NAD^+^ supplementation restores osteoclast differentiation, confirming that NAD^+^ insufficiency directly inhibits osteoclast activity. In contrast, MAS inhibitor treatment reduced ATP and NAD^+^ levels and reversed the pro-osteoclastogenic effect of FSH, further suggesting that NAD^+^ is the key to FSH regulation of energy homeostasis [34]. Furthermore, researchers found that Mdh2 expression is up-regulated during osteoclast differentiation, and its knockdown inhibits osteoclast activity and energy metabolism [34]. Chromatin immunoprecipitation and luciferase assays confirmed that FSH phosphorylates cAMP response element-binding protein (CREB), which promotes its binding to the Mdh2 promoter and enhances its transcription [34]. Therefore, the effect of FSH on osteoclasts is dependent on Mdh2 through the mechanism by which FSH increases CREB phosphorylation, upregulates Mdh2 expression, and promotes MAS and NAD^+^ regeneration. In vivo experiments in OVX mice have also validated the role of NAD^+^ metabolism in regulating bone resorption with FSH [34]. The study demonstrated that osteoclast energy crisis is a central factor in the imbalance of bone homeostasis, and that FSH drives metabolic reprogramming through the CREB-MDH2-NAD^+^ axis, leading to the over-activation of osteoclasts, which in turn disrupts bone homeostasis and exacerbates osteoporosis [34]. This study not only deepens the understanding of energy homeostasis in bone metabolism but also provides a molecular basis for clinical interventions and emphasises that targeting energy metabolism could be a new strategy for treating bone loss diseases.

Notably, rather than serving merely as a passive reservoir for minerals and lipid storage (e.g., within bone marrow adipocytes), bone actively participates in the regulation of whole-body energy metabolism through the secretion of multiple osteokines [29,35]. Conversely, the whole-body energy metabolism significantly influences bone health. For example, studies on the relationship between lactation duration and risk of vertebral fractures suggest that changes in calcium metabolism during lactation may contribute to bone loss [36]. In addition, whole blood viscosity is negatively correlated with bone mineral density in post-menopausal osteoporotic women [37]. The nervous system also regulates energy and bone homeostasis through, for example, the synaptic adhesion molecule Calsyntenin-3 [38]. In conclusion, the energy sensing network (especially the AMPK and NAD^+^ metabolic axis) reprograms the cellular metabolic pattern and becomes a key hub connecting energy imbalance and bone homeostasis disorder. Targeting the signaling pathways such as GLP-1R–AMPK, IMP2–LKB1–AMPK, and FSH–CREB–MDH2–NAD^+^, it is expected to reverse the imbalance between bone formation and resorption from the metabolic root, providing a new treatment strategy for metabolic bone diseases.

### 3.2. Metabolite Signalling: Chemical Messengers in the Bone Microenvironment

Metabolites in the bone microenvironment play a key role in chronic bone diseases by influencing bone formation, resorption, and remodelling through a variety of mechanisms [39,40]. A wide variety of metabolites, including amino acids, lipids, glucose, and their derivatives, can modulate osteoblast function and directly or indirectly influence the inflammatory state of the bone microenvironment [39,40]. Patel et al. [41] explored the effect of age on bone tissue metabolites in C57BL/6 mice and found a clear separation among the metabolite profiles of young and old mice, suggesting that age is a major driver of changes in bone metabolism. A variety of metabolites were consistently down-regulated in male and female aged mice, including 4-hydroxyproline, glutamine, and α-linolenic acid [41]. These metabolites are associated with bone integrity; for example, the decline in hydroxyproline, a key component of collagen, may reflect increased collagen degradation, and its reduction may weaken the stability of the bone matrix [41]. Therefore, bone metabolites change significantly with age and may be associated with age-related bone loss, suggesting that targeting these metabolites may help ameliorate a variety of skeletal disorders caused by aging. Furthermore, in patients with chronic kidney disease-mineral bone disease, an imbalance in central nervous system tryptophan-kynurenine metabolism has also been associated with increased bone fragility, suggesting that this pathway may remotely regulate bone health through the ‘brain-bone axis’ [42]. Diabetic osteoporosis is a classic example of a metabolite-driven chronic bone disease. In a db/db diabetic mouse model, Yang et al. [43] found that the level of lipid peroxidation in osteoblasts is significantly elevated, and the use of ferrostatin-1, an iron death inhibitor, effectively rescues the loss of bone mass, demonstrating that iron death is a key mechanism linking metabolic disorders to osteoblast death. Furthermore, metabolomics studies by Wu et al. [44] discovered that bone metabolism disorders in type 2 diabetic osteoporosis (T2DOP) are centred on the dysregulation of lipid and amino acid metabolisms; especially, fatty acid accumulation and disruption of the glutathione pathway drive bone loss. Moreover, these metabolite changes are time-dependent, with predominantly early lipid abnormalities and late intensification of amino acid metabolism [44]. These findings provide potential biomarkers for T2DOP. Therefore, targeting specific metabolites and pathways may be beneficial for improving bone health. In addition, some studies have confirmed that different secondary metabolites of turmeric have different mechanisms of osteoprotective effects in different skeletal disease models, suggesting that metabolites from traditional medicinal plant sources have diverse pharmacodynamic targets and exert multi-targeted effects in the treatment of various skeletal diseases [45].

Studies have demonstrated that L-arginine, a naturally occurring amino acid, significantly inhibits osteoclastogenesis in a dose-dependent manner, as evidenced by the low expression of osteoclast-specific genes (such as *Traf6*, *Acp5*, *Nfatc1*, *Ctsk*, and *Atp6v0d2*) [46]. Kyoto Encyclopaedia of Genes and Genomes (KEGG) pathway analyses demonstrated that, when osteoclasts are stimulated by TNF-α, L-arginine treatment raises oxidative phosphorylation pathway gene expression levels [46]. Further experiments revealed that TNF-α stimulation promotes glycolysis and inhibits oxidative phosphorylation in osteoclasts, thereby accelerating osteoclastogenesis, which may be further reversed by L-arginine [46]. These results suggest that L-arginine inhibits inflammation-induced bone loss by reprogramming osteoclast metabolic processes. Therefore, L-arginine supplementation may be a potential strategy to protect against inflammatory bone loss. Similarly, aconitic acid decarboxylase 1 (ACOD1) and its metabolite, itaconic acid, have been shown to be key regulators of osteoclast differentiation and bone loss in inflammatory arthritis [47].

In conclusion, metabolites in the bone microenvironment are not merely passive reflections of disease states but also active participants and potential therapeutic targets. Targeting key metabolic nodes such as L-arginine and itaconic acid to reprogram osteoclast energy metabolism, or interfering with ferroptosis and lipid oxidation pathways, holds promise for restoring bone homeostasis from a metabolic perspective and providing a new paradigm for precise intervention in chronic bone diseases. Future research should further elucidate the dynamic changes of specific metabolites in the spatiotemporal dimension, clarify their mechanisms of action in different chronic bone diseases, and explore new approaches to reshape the bone metabolic microenvironment through dietary intervention, probiotic regulation, metabolic enzyme inhibitors, or precursor substance supplementation.

## 4. Chronic Bone Disease: Pivotal Role of Metabolic Reprogramming in Macrophage Polarisation

### 4.1. Chronic Bone Disease: Mechanisms of Interaction Between Glucose Metabolism and Macrophage Polarisation

Macrophages are highly plastic and can differentiate into different phenotypes in response to microenvironmental stimuli [48]. Recent studies have revealed that metabolic pathways are not only the result of polarisation but also of its drivers [49]. M1-type polarisation relies on glycolysis for rapid energy provision, whereas M2-type macrophages tend to use oxidative phosphorylation to support long-term repair. In vitro experiments have confirmed that fluorinated porcine hydroxyapatite (FPHA) extracts inhibit M1-type polarisation induced by LPS, as evidenced by the down-regulation of inducible nitric oxide synthase (iNOS) (a key M1 marker), both at the gene and protein levels, and a reduction in nitric oxide release [50]. Furthermore, FPHA promotes M2-type polarisation, resulting in the up-regulation of the expression of the M2-associated marker Arg. RNA-seq analyses revealed that the polarisation-regulating function of FPHA is closely related to cellular metabolic reprogramming. Besides, a gene set enrichment analysis showed that cells cultured with FPHA extracts had up-regulated oxidative phosphorylation and gluconeogenesis pathways [50]. Further metabolic function experiments confirmed these findings; oxidative phosphorylation parameters such as basal respiration, maximal respiratory capacity, and ATP production were significantly elevated in macrophages from the FPHA group after 6 h of incubation, whereas the glycolytic capacity and reserve were significantly suppressed after 1 day of incubation [50]. This metabolic shift from glycolysis (M1-associated) to oxidative phosphorylation (M2-associated) is a key mechanism driving macrophage polarisation towards the M2 phenotype. In vivo rat cranial defect model experiments further verified that, on day 7 after FPHA implantation, the defect area showed increased M2-type macrophage infiltration and up-regulation of succinate dehydrogenase B expression, confirming that FPHA induces M2 polarisation by promoting oxidative phosphorylation [50]. Thus, this macrophage polarisation process is closely related to the reprogramming of cellular energy metabolism, particularly the alteration of glycolytic pathways [51,52]. In a variety of chronic bone diseases, dysregulation of glucose metabolism directly affects osteoblast status and impedes bone regeneration and remodelling, exacerbating disease development. For example, a study by Ying et al. [53] demonstrated that, in osteoarthritis (OA), the interleukin-1β (IL-1β)-mediated inflammatory response significantly upregulates the expression of pyruvate dehydrogenase subunit E1α and lactate dehydrogenase A (LDHA) in pre-osteoblasts, and dose-dependently enhances the levels of oxidative phosphorylation and glycolysis, whilst simultaneously increasing intracellular ATP synthesis. This metabolic reprogramming directly promotes abnormal differentiation and excessive osteogenesis in pre-osteoblasts, ultimately leading to subchondral bone sclerosis [53]. Notably, inhibiting glycolysis with 2-deoxy-D-glucose or inhibiting oxidative phosphorylation with oligomycin effectively blocks the IL-1β-induced upregulation of osteogenic markers (such as OCN, OPN, ALP and Osterix), mitigates abnormal subchondral bone remodelling, and protects articular cartilage from degradation [53]. These findings reveal the potential therapeutic value of targeting glucose metabolic pathways in intervening in the progression of OA. In addition, chronic hyperglycaemia not only directly affects osteoblast function but may also indirectly impair bone health by affecting macrophage polarisation [54,55]. For example, the accumulation of advanced glycosylation end products (AGEs) under hyperglycaemic conditions can inhibit osteoclastogenesis and enhance osteoblastogenesis, ultimately leading to increased bone resorption [56]. The accumulation of AGEs has also been found to promote macrophage polarisation towards M1 type through the HIF-1α/PDK4 pathway [57]. In addition, sirtuin-1 regulates glucose metabolism and maintains glucose homeostasis in various metabolic tissues [58]. In diabetic osteoporosis, the nanomedicine ‘Tsa’ acts as a gene-activating tetrahedral nucleic acid that up-regulates sirtuin-1 gene expression and shifts macrophage polarisation to an anti-inflammatory M2 phenotype, creating a favourable skeletal immune microenvironment for osteoblasts [59].

**Figure 2:**
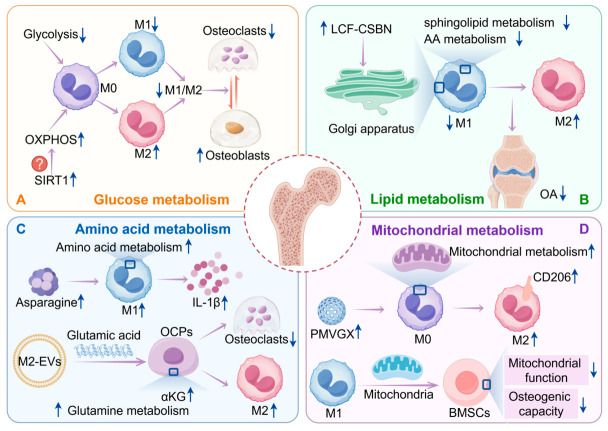
The pivotal role of metabolic reprogramming in driving macrophage polarisation and regulating bone homeostasis during chronic bone disorders. This schematic illustrates how distinct metabolic pathways determine macrophage phenotypic shifts, thereby influencing bone remodelling and disease progression, specifically highlighting the reprogramming of these pathways using the action of specific experimental drugs and agents as illustrative examples. (**A**) Glucose metabolism: Metabolic switching from glycolysis to oxidative phosphorylation drives macrophages from pro-inflammatory M1 to anti-inflammatory M2 phenotypes. Upregulation of sirtuin-1 (SIRT1) expression may facilitate this transition, thereby inhibiting osteoclast activity and promoting osteoblast function. (**B**) Lipid metabolism: Exemplified by licofelone-loaded nanoparticle (termed LCF-CSBN) targeting the Golgi apparatus, which promote M1-to-M2 phenotype conversion by inhibiting sphingolipid and arachidonic acid (AA) metabolism, thereby alleviating osteoarthritis (OA) progression. (**C**) Amino acid metabolism: Elevated asparagine levels drive M1 polarisation and interleukin-1β (IL-1β) secretion. Conversely, M2-type macrophage-derived extracellular vesicles (M2-EVs) deliver glutamate to osteoclast precursors (OCPs), enhancing glutamine metabolism and α-ketoglutarate production. This metabolic reprogramming induces OCPs’ M2 phenotype shift and inhibits osteoclastogenesis. (**D**) Mitochondrial metabolism: Agents such as VGX-1027 microcarrier (PMVGX) drive M2 polarisation by enhancing mitochondrial metabolism. Conversely, mitochondrial transfer from M1 macrophages to bone marrow mesenchymal stem cells (BMSCs) impairs mitochondrial function and inhibits their osteogenic capacity. Note: The dark blue upward and downward-pointing arrows indicate the upregulation and downregulation of the corresponding targets, biomarkers, or biological processes, respectively. This figure was generated using Figdraw (www.figdraw.com), with the copyright code SPWUTc8338.

Targeting the sirtuin-1 gene may have great potential to modulate skeletal disorders due to glucose metabolism imbalance by altering the macrophage polarisation status through the regulation of glucose metabolism. In summary, the metabolic reprogramming of macrophages is the key switch that regulates the bone immune microenvironment. Targeting the glycolytic pathway (such as sirtuin-1) or reshaping the balance between oxidative phosphorylation and glycolysis can drive the phenotypic change of macrophages (Figure 2), thereby reversing the bone homeostatic imbalance driven by high glucose and inflammation, and is expected to provide a new metabolic intervention-based immunomodulatory strategy for chronic bone diseases [6,60].

### 4.2. Chronic Bone Disease: Mechanisms of Interaction Between Lipid Metabolism and Macrophage Polarisation

In recent years, the association between lipid and bone metabolism has received widespread attention. Studies have confirmed that lipid metabolism not only plays a key role in the maintenance of bone homeostasis but is also one of the central drivers of disease progression in pathological states, such as osteoporosis and OA [61]. For example, Lin et al. [62] observed that bone marrow adipocyte accumulation and lipid metabolism are significantly increased in the bone marrow of mice modelled for osteoporosis. Studies have also demonstrated that polyunsaturated fatty acids in the bone marrow promote bone marrow haematopoietic stem cell adipogenesis and exacerbate mitochondrial dysfunction in osteoblasts [62], suggesting that excess lipids lead to impaired osteogenic capacity, which in turn promotes the development of bone diseases. In addition, a strong link between bone metabolism disorders and adipose tissue has been revealed through in vivo and in vitro experiments. In a high-fat diet (HFD)-induced obese mouse model, epithelial white adipose tissue (eWAT)-derived osteoblastic protein (OPN) has shown a central role in obesity-associated bone metabolism disorders [63]. Specifically, eWAT macrophages are the main source of OPN, and OPN secretion is increased under HFD and is selectively targeted to the bone marrow through blood circulation [63]. OPN can promote osteoclast-mediated bone resorption and lipid metabolism, but, at the same time, OPN-induced lactate accumulation can feed back to inhibit bone loss and lipolysis [63]. These experimental results not only suggest that lipid metabolism has a profound effect on bone health but also validate the possibility of regulating bone homeostasis through macrophage secretion. Importantly, macrophage M2-type polarisation is closely related to lipid metabolism [64]. Xu et al. [64] revealed through in vitro experiments that arachidonic acid metabolism plays a key role in IL-4/IL-13-induced M2-type polarisation in macrophages; mechanistically, the arachidonic acid-derived metabolite prostaglandin E2 is able to augment oxidative phosphorylation through the inhibition of PPARγ, which, in turn, promotes macrophage M2-type polarisation. Therefore, lipids regulate macrophage activation and polarisation by acting as signalling molecules, thereby shaping the immune response [65]. Notably, in recent years, many researchers have focused on whether macrophage polarisation can be regulated through the reprogramming of lipid metabolism, which in turn can influence the disease process, or used in the development of novel therapeutic targets and drugs [66]. This mechanism has received similar attention for the treatment of chronic bone diseases. For example, some researchers have developed licofelone-loaded nanoparticle (termed LCF-CSBN), which can alleviate the symptoms of OA by reprogramming lipid metabolism in M1-type macrophages [67]. LCF-CSBN was confirmed to be effective in reducing reactive oxygen species levels and Golgi stress in M1-type macrophages, which positively correlates with OA synovial inflammation, suggesting that LCF-CSBN has the potential to alleviate OA symptoms [67]. In addition, targeted liposome analysis and a KEGG analysis revealed that LCF-CSBN exerted a dramatic effect on the reprogramming of ceramide and arachidonic acid metabolism in M1 macrophages [67]. Further experiments confirmed that LCF-CSBN enhances the repolarisation of M1-type macrophages to the M2 phenotype by reprogramming its lipid metabolism and inhibiting its pro-inflammatory signalling pathways [67]. In OA model rats, LCF-CSBN also exhibits significant and long-lasting anti-inflammatory and analgesic effects as well as amelioration of cartilage degeneration, suggesting that this nanoparticle can slow down the progression of OA [67]. Therefore, altering macrophage phenotypes by reprogramming lipid metabolism may be a promising therapeutic strategy to delay the progression of OA, suggesting that this mechanism may play an equivalent role in similar chronic bone diseases.

In summary, targeting the lipid metabolism-macrophage axis is expected to provide a novel therapeutic strategy for chronic bone diseases (Figure 2). However, this interaction network may vary across chronic bone diseases and at different disease stages, requiring further refined studies on the underlying spatio-temporal dynamics. In the future, the integration of single-cell sequencing, spatial metabolomics, and in vivo imaging technologies will provide a clearer picture of how lipids in the bone marrow or joint cavity specifically affect macrophage subpopulations and how the latter communicate metabolically via vectors such as exosomes. The development of synergistic therapies targeting the bone immunometabolic microenvironment based on these mechanisms is expected to provide new treatment options for patients with chronic bone diseases who have had poor results or are refractory to conventional therapies.

### 4.3. Chronic Bone Disease: Mechanism of Interaction Between Amino Acid Metabolism and Macrophage Polarisation

In recent years, it has been found that amino acids are not only components of proteins, but their related metabolic pathways can also provide energy for macrophage polarisation, which plays an important role in the regulation of macrophage function [68]. Studies have revealed the central role of glutamine metabolism in determining macrophage polarisation fate. For example, a study by Long et al. [69] demonstrated that the glutamine-glutamate/γ-aminobutyric acid metabolic cycle is a key immunometabolic hub for regulating macrophage functional polarization and alleviating rheumatoid arthritis. The study hypothesised that glutamine is sequentially converted into glutamate and γ-aminobutyric acid by the action of glutaminase and glutamate decarboxylase [69]. The upregulated γ-aminobutyric acid may increase prolyl hydroxylase domain-dependent IκB kinase β hydroxylation modifications through the succinate pathway, which accompanies the tricarboxylic acid cycle (TCA cycle), thereby limiting the activation of M1 macrophages [69]. At the same time, glutamate dehydrogenase 2, a key potential target in this process, catalyses the conversion of glutamate into α-ketoglutarate [69]. The study speculated that changes in α-ketoglutarate levels could inhibit IκB kinase β activity, blocking the signal transduction of the NF-κB complex, and thus suppressing the proliferation of pro-inflammatory M1 macrophages and the release of inflammatory mediators such as IL-1α, IL-1β, and MMP3 [69]. Metabolomics analyses by Huang et al. [70] revealed that M2-type macrophage-derived extracellular vesicles (M2-EVs) were significantly enriched in glutamine compared to M0-EVs. When M2-EVs were internalised by osteoclast precursors (OCPs), the glutamate they carried was delivered to the cells and became the ‘metabolic signalling’ starting point, and glutamate was further catalysed by the key enzyme glutamate dehydrogenase to produce alpha-ketoglutarate (αKG), which was manifested by the increased activity of glutamate dehydrogenase and αKG itself. Notably, the intracellular levels of αKG were significantly increased [70]. These results confirmed the enhancing effect of M2-EVs on glutamine metabolism within OCPs. To further validate the functional importance of this pathway, the glutamine metabolism inhibitor R162 was used. The results showed that R162 treatment effectively reversed the M2-EVs-induced transition of OCPs to an M2-like phenotype, whereas supplementation with exogenous dimethyl (DM)-αKG mimicked the effect of M2-EVs [70]. This ‘inhibition-rescue’ experiment provides strong evidence that activation of glutamine metabolism is necessary for the polarisation of OCPs into M2-like macrophages. In addition, the metabolite αKG can serve as a bridge for epigenetic regulation to drive the polarisation shift. αKG is not only an intermediate in the tricarboxylic acid cycle but also an essential cofactor for a variety of epigenetically modified enzymes. It was confirmed that the αKG-driven histone demethylase (Jumonji domain containing-3, Jmjd3) activity was enhanced in M2-EVs-treated OCPs, leading to enhanced demethylation of the repressive histone 3 lysine 27 trimethylation (H3K27me3) mark [70]. Chromatin immunoprecipitation experiments confirmed that M2-EVs treatment resulted in an enrichment of demethylated H3K27me3 in the promoter regions of osteoclast genes (e.g., *Nfatc1* and *Mmp9*) and decreased levels of demethylated H3K27me3 in the promoter regions of M2 genes (e.g., *Retnia* and *Mrc1*) [70], which effectively down-regulated the expression of osteoclast-specific genes and inhibited osteoclast differentiation. This metabolite-driven epigenetic remodelling, which directly links the intracellular metabolic state to gene expression, is the central molecular mechanism through which OCPs abandon their osteoclast fate and shift to an M2-like macrophage-polarised state. This mechanism profoundly reveals that metabolism is not only a consequence of cellular polarisation but also an active factor driving the onset of polarisation. Metabolic intermediates (e.g., αKG) act as key switches in determining cell fate by modulating epigenetics, providing a new theoretical basis for understanding the role of immune metabolism in skeletal homeostasis. Similarly, Zheng et al. [71] found that leucine, as an essential branched-chain amino acid, activates liver X receptor α signalling via a mechanism dependent on mammalian target of rapamycin complex 1, thereby precisely regulating metabolic reprogramming in macrophages. Leucine promotes the nuclear translocation of liver X receptor α and enhances its transcriptional activity, driving the reprogramming of synovial macrophages into an M2-like state, significantly suppressing the secretion of inflammatory cytokines such as TNF-α and IL-1β, and other inflammatory factors, whilst simultaneously upregulating the expression levels of markers such as Arg1 and IL-10 [71]. Further studies revealed that this process blocks the abnormal activation of the β-catenin signalling pathway within chondrocyte precursors by reducing the paracrine release of R-spondin 2 from M1 macrophages, thereby maintaining the secretory capacity of the chondrocyte extracellular matrix and delaying the degeneration of articular cartilage and the formation of osteophytes [71]. The elucidation of the aforementioned ‘metabolic-immune-cartilage’ regulatory axis redefines nutritional amino acids as immunometabolic modulators in degenerative joint diseases; in particular, this axis provides new molecular evidence supporting leucine supplementation as an effective strategy for disrupting the inflammation-bone remodelling cycle in traumatic arthritis.

Experimental studies by Yang et al. [72] have shown that aminooxyacetic acid hemihydrochloride, acting as an inhibitor of the MAS, can reduce the level of mitochondrial oxidative phosphorylation by blocking the exchange of reducing equivalents between the cytosol and the mitochondrial matrix. This effect results in a reduction in ATP production and a decrease in reactive oxygen species generation within osteoclasts, thereby effectively inhibiting osteoclast differentiation and their bone resorption function [72]. In an ovariectomy-induced osteoporosis mouse model, aminooxyacetic acid hemihydrochloride treatment significantly alleviated bone mass loss and improved bone microstructure parameters [72]. These findings suggest that targeting aspartate-related metabolic pathways may offer new intervention strategies for the prevention and treatment of bone diseases. In addition, aspartate metabolism exhibits significant changes during M1-type macrophage polarisation [73]. Further experiments revealed that aspartate and its derivative asparagine were able to promote M1 macrophage polarisation and secretion of IL-1β, which was mainly dependent on metabolic remodelling as well as activation of HIF-1α and NLRP3 inflammatory vesicles [73]. Although this study did not focus directly on skeletal disorders, IL-1β is a key inflammatory factor driving osteoclast differentiation in rheumatoid arthritis and osteoporosis, providing a fundamental mechanism for understanding the role of aspartate metabolism in immune-mediated bone diseases. However, research into how aspartate metabolism regulates macrophages is still in its early stages. Its direct interaction with the skeletal immune microenvironment, as well as its specific application as a therapeutic target for disease, remains an important area requiring further exploration.

In conclusion, the metabolism of amino acids such as glutamine, leucine, and aspartic acid is not only the result of macrophage polarization, but also the core mechanism that drives the phenotypic transformation and regulates the bone immune microenvironment (Figure 2). In-depth analysis of the molecular network of the “metabolism-immunity-bone/cartilage” axis, especially further exploration of direct interaction mechanisms such as aspartic acid metabolism that are still in the initial stage, will provide highly promising new strategies and new targets for targeted immunometabolic therapy of chronic bone diseases such as rheumatoid arthritis and osteoporosis.

### 4.4. Chronic Bone Disease: Mechanisms of Interaction Between Mitochondrial Metabolism and Macrophage Polarisation

The link between mitochondrial metabolism and macrophages has received considerable attention in recent years. Studies have confirmed that, in LPS-activated macrophages, the reprogramming of mitochondrial metabolism is the key to driving macrophage inflammatory function [74]. In chronic bone diseases, there is a complex interaction between mitochondrial metabolism and macrophage polarisation that has important implications for disease development and therapeutic interventions [75]. Wang et al. [76] reported the development of a microcarrier loaded with the immunomodulator VGX-1027 (PMVGX), which was able to repair femoral bone defects and attenuate inflammation in the osteoporotic bone tissue of OVX mice. In addition, cellular experimental data showed that the expression of mitochondrial activity-related proteins (such as mitochondrial fusion protein 2, optic atrophy protein 1, and dynamin-related protein 1) was significantly up-regulated in macrophages after treatment with PMVGX [76]. More importantly, both the cellular oxygen consumption rate and intracellular ATP content were significantly increased, indicating that PMVGX enhances mitochondrial oxidative phosphorylation activity and elevates cellular energy metabolism. Besides, enhanced mitochondrial metabolism provides the necessary energy support and metabolic basis for macrophage polarisation towards the M2 phenotype. The study confirmed that PMVGX-induced mitochondrial activation occurred in parallel with the up-regulation of M2 polarisation markers (e.g., CD206), the increase in the secretion of anti-inflammatory factors (up-regulation of anti-inflammatory factors, such as IL-10, TGF-β, and vascular endothelial growth factor [VEGF]), and the inhibition of pro-inflammatory factors (such as TNF-α and IL-1α) [76]. This suggests that the mitochondrial energy supply triggered by PMVGX is a key driver of the successful transformation of macrophages into a reparative M2 phenotype. Furthermore, enhanced mitochondrial metabolism directly supports another key function, efferocytosis (also known as cytoburial). Cytoburial is an active process involving cytoskeletal remodelling and phagocytic vesicle formation and transport; it consumes large amounts of ATP. In the study, it was observed that macrophage clearance of apoptotic cells was significantly enhanced after PMVGX treatment, accompanied by a rise in the expression of cytoburial-associated proteins, such as e.g., platelet reactive protein-1, CD36, and C-Mer proto-oncogene tyrosine kinase [76]. This suggests that mitochondria activated by PMVGX provide sufficient power for the energy-consuming cytoburial process. Therefore, mitochondrial metabolic reprogramming shapes the anti-inflammatory bone immune microenvironment by promoting M2 polarisation and cytoburial; this benign microenvironment is the core of the bone immunomodulatory function of PMVGX microcarriers. It is worth noting that M’baya-Moutoula et al. [77] found, through multi-omics analysis, that miR-223 regulates several genes associated with the mitochondrial respiratory chain in the RAW 264.7 monocyte/macrophage cell line, such as Ndufaf4 and Ndufaf6, which are components of mitochondrial complex I. Complex I plays a central role in the balance between cell proliferation and apoptosis; its abnormal accumulation can induce reactive oxygen species production and cell death [77]. The study further demonstrated that both the overexpression and inhibition of miR-223 significantly alter the levels of antioxidant metabolites, such as glutathione and cysteine, within macrophages, suggesting that miR-223 influences oxidative stress and survival in macrophages by regulating mitochondrial metabolic status [77]. Furthermore, this miRNA directly targets CARM-1 (co-activator-related arginine methyltransferase 1) and regulates the expression of HDAC5 (histone deacetylase 5). The former catalyses the methylation of histone H3 and participates in the positive regulation of the NF-κB signalling pathway; the latter negatively regulates osteogenesis by deacetylating runt-related transcription factor 2 (RUNX2) [77]. During osteoclast differentiation, upregulation of miR-223 significantly increases the expression of genes such as *Cactin*, *CARM-1* and *MPC-1*, accompanied by elevated NF-κB levels. Furthermore, in monocyte/macrophage cell lines polarising towards M1 macrophages, miR-223 downregulates NF-κB expression; conversely, during M2 polarisation, it upregulates NF-κB expression [77]. These results indicate that miR-223, as a key node in the epigenetic regulatory network, influences the polarisation of macrophages towards the M1/M2 or osteoclast lineages by integrating mitochondrial metabolic reprogramming (such as alterations in complex I components and glutathione metabolism) with histone modifications (Hdac5, CARM-1) [77]. Consequently, the underlying mechanisms governing the dynamic changes in macrophages regulated by mitochondrial metabolism may lie in epigenetic or non-coding RNA alterations, which ultimately profoundly determine global bone homeostasis in chronic disease states. In addition, Cai et al. [78] demonstrated that the percentage of macrophages with the M1 phenotype was significantly increased in OVX osteoporotic mice, whereas the percentage of M2 phenotype was significantly decreased, suggesting that macrophages tend to differentiate towards the M1 phenotype in osteoporotic disease states. Furthermore, experimental results have shown that MSCs receiving macrophage-derived mitochondria from OVX mice have reduced osteogenic capacity, whereas MSCs receiving normal-functioning mitochondria show a stronger osteogenic phenotype [78]. Further experiments revealed that the mitochondria of M1 macrophages induced by LPS treatment influence the metabolic state of MSCs, as evidenced by impaired mitochondrial function and enhanced glycolytic activity [78]. Therefore, macrophages can promote metabolic remodelling by transferring mitochondria to bone marrow MSCs, which in turn regulate bone homeostasis in osteoporosis.

In summary, mitochondrial metabolism is not only a determinant of macrophage polarisation but also a key node connecting immune regulation and bone metabolism, which has important translational medical value (Figure 2). A detailed analysis of the crosstalk mechanism between mitochondrial metabolism and epigenetic networks/non-coding RNAs is expected to provide highly promising innovative strategies for the development of new biomaterials and targeted metabolic therapies for chronic bone diseases.

### 4.5. Chronic Bone Disease: Metabolites That Modulate Macrophage Polarisation

#### 4.5.1. Succinic Acid

In recent years, succinate, an important intermediate metabolite of the tricarboxylic acid cycle, has been shown to be involved in cellular energy metabolism and to play a key role as a signalling molecule in various bone diseases [79]. For example, in a mouse model of type 2 diabetes, succinate levels are elevated and signal through the succinate receptor 1 (SUCNR1) in osteoclasts, thereby inducing osteoclastogenesis and promoting bone resorption [80]. Similarly, a study by Chen et al. [81] explored the specific mechanism by which curcumenol exerts its therapeutic effects on knee osteoarthritis, confirming that this compound up-regulates the expression of the histone demethylase KDM6B, inhibits the methylation of H3K27, and decreases the expression of downstream metabolites such as succinate, which improves the metabolic homeostasis of knee osteoarthritis cartilage. Notably, succinic acid has also been shown to promote bone tissue regeneration in bone healing models, and in vitro experiments have revealed that extracellular succinic acid enhances osteogenic differentiation and angiogenic and migratory processes in primary human cells [82]. These findings suggest that succinic acid can exhibit two opposite effects, pro-osteoblastic and pro-osteogenic, in the regulation of bone metabolism, which may be related to the physiological state of the organism or tissue in which it is found.

In addition, Xu et al. [83] observed that alveolar bone loss in old mice is accompanied by an increase in succinate content, suggesting that succinate and its receptor may be involved in bone health during aging. Further experiments confirmed that deletion of SUCNR1 was effective in reducing aging-associated succinate elevation as well as attenuating age-related inflammation and bone loss, as evidenced by a significant reduction in the levels of pro-inflammatory cytokines, including TNF-α, keratinocyte-derived chemokine/growth-regulated oncogene, interleukin-6 (IL-6), interleukin-2, and interferon-gamma as well as significant increases in bone mineral density, bone volume, and the bone volume/total volume ratio [83]. Therefore, succinate and its receptor SUCNR1 not only play a driving role in the process of bone loss but may also be involved in the inflammatory response and immune regulation. Furthermore, it has been demonstrated that type I Interferon-beta (IFN-β) promotes succinate accumulation and reduces αKG, i.e., IFN-β reduces the αKG/succinate ratio [84]. Further experiments confirmed that IFN-β inhibits IL-4-induced macrophage M2 polarisation by down-regulating the αKG/succinate ratio [84]. Therefore, succinate can modulate the functional phenotype of macrophages, i.e., inhibits M2 polarisation. Notably, succinic acid may also be involved in inducing M2 polarisation under certain circumstances. For example, in an ocular neovascularization model, succinic acid induces macrophages to secrete retinol-binding protein 4 and induces M2 polarisation via SUCNR1, thereby contributing to an increase in the M2/M1 ratio [85]. However, an increase in this ratio does not necessarily trigger a positive effect but may promote vascular endothelial cell migration, invasion, and canalisation, thereby promoting pathological angiogenesis [85]. However, in most chronic bone diseases, succinate primarily drives persistent low-grade inflammation and disrupts bone homeostasis [78]. The mechanisms mainly involve succinate receptor SUCNR1-mediated signalling pathway activation, metabolic reprogramming, and interactions with other immune cells and osteoblasts [86]. Collectively, these findings emphasise the importance of targeting succinate and its receptor to influence the macrophage phenotype and activity. However, conflicting findings from existing studies suggest that the function of the metabolite succinic acid in the bone microenvironment is highly context-dependent, with its effects influenced by various factors such as cell type, tissue environment, disease state, metabolite concentration and form. Furthermore, the mechanisms by which the interaction between succinic acid and macrophage polarisation influences the balance between inflammatory responses and bone remodelling within the bone microenvironment remain unclear, and further research is required to elucidate this complex mechanism.

#### 4.5.2. Itaconic Acid

Itaconate is a metabolite with immunomodulatory activity that is catalysed by ACOD1 in macrophage mitochondria. Recent studies have shown that itaconate, an endogenous metabolite, plays a key role in the regulation of bone metabolism and macrophage function [87,88,89]. In vivo experiments by Kieler et al. [89] have demonstrated that itaconate can stimulate osteoblast maturation, and in vitro experiments have demonstrated that itaconate can up-regulate the expression of osteoblast-related genes (such as *ALP*, *RUNX2*, *BSP*, *OPN*, and *COL1A1*) and increase the formation of bone nodules and calcium deposition [89], suggesting that itaconate can act as an osteoclast regulatory factor. Another study by Rong et al. [90] found that itaconic acid produced by inflammatory macrophages may play a role in the inhibition of osteoclast formation in a paracrine manner, suggesting that it plays a regulatory role in bone metabolism. In addition, cellular experiments have revealed that treatment with an itaconic acid derivative, exogenous 4-octyl clathrate (OI), significantly reduces the proportion of CD86 cells and the M1/M2 ratio, suggesting that OI induces partial conversion of M1 macrophages to the M2 phenotype [90]. Moreover, in vivo experiments have confirmed the protective effect of exogenously administered itaconic acid and its derivative OI in rheumatoid arthritis without significant tissue toxicity [90]. These experimental results suggest that itaconic acid and its derivatives have great potential as therapeutic agents for a variety of bone diseases, and that this metabolite may influence the development of a variety of chronic bone diseases by modulating macrophage polarisation.

Furthermore, An et al. [91] revealed that itaconic acid plays a key immunomodulatory role in steroid-associated osteonecrosis (SAON). This study was the first to demonstrate that hydrogen-rich water could effectively improve SAON by promoting osteogenesis, decreasing bone resorption, promoting angiogenesis, and improving lipid metabolism [91]. Further analysis of bone tissue from mice with SAON, using RNA-seq, revealed that the expression of ACOD1 was significantly up-regulated in a hydrogen-rich water-treated group, and the C5-branched dicarboxylic acid metabolic pathway to which it belonged was the top-ranked enriched pathway [91]. This finding was also confirmed at the mRNA level; that is, ACOD1 expression was significantly decreased in the SAON model group, whereas hydrogen-enriched water intervention restored it to near-normal levels, suggesting that the bone-beneficial effect of hydrogen-rich water may be achieved by up-regulating ACOD1. Critically, after using dimethyl itaconate, an analogue of itaconic acid, to treat mice with SAON, researchers found that dimethyl itaconate treatment reversed the decrease in ACOD1 levels in the SAON model group and reproduced the bone-protective effects of hydrogen-rich water in a dose-dependent manner [91]. Further, dimethyl itaconate treatment not only significantly reduced the expression of the M1 macrophage marker CD86 and elevated the expression of the M2 marker CD206 in bone tissue, but also synchronously suppressed the level of the key pro-inflammatory factor TNF-α [91]. It has been further suggested that macrophage polarisation mediated by ACOD1 and its metabolite, itaconic acid, might be key to the treatment of hormonal osteonecrosis. The activation of the ACOD1-itaconic acid metabolic axis effectively reversed the chronic inflammatory state in SAON, which provided a solid experimental rationale and a new intervention strategy for targeting immunometabolism in the treatment of chronic bone diseases. Notably, itaconic acid plays a dual role in macrophage polarisation. It is a key inhibitor of inflammatory signalling pathways and exerts anti-inflammatory effects through multiple mechanisms [87]. Recent studies by Runtsch et al. [92] have revealed that itaconic acid and its derivatives inhibit alternative activation and metabolic remodelling of M2 macrophages. In addition, itaconic acid and OI block IL-4 signalling by inhibiting the phosphorylation of Janus kinase 1 and signal transducer and activator of transcription 6, thereby inhibiting the polarisation of M2 macrophages [92]. This suggests that itaconic acid has a complex balancing effect on immunomodulation, inhibiting the pro-inflammatory response of the M1 type macrophages and modulating the anti-inflammatory/repairing response of the M2 type macrophages under specific circumstances. Therefore, balancing the regulatory effects of itaconic acid on M1 and M2 macrophage polarisation to achieve the best therapeutic effect is an important direction for future research.

In summary, itaconic acid is a key node connecting immune metabolism and chronic bone diseases through the multi-target regulation of macrophage polarisation. Future studies are needed to further clarify the spatiotemporal-specific mechanism of action of itaconic acid in different types of bone diseases and to explore targeted delivery strategies based on itaconic acid derivatives to achieve more precise immunometabolic interventions.

#### 4.5.3. Citric Acid/Fumaric Acid

Citric acid plays a central role in cellular energy metabolism and signalling regulation as a key metabolic intermediate in the TCA cycle [93]. Recent studies have shown that organic acids not only participate in basic biological oxidation processes but may also play an important role in the development of chronic bone diseases by regulating the metabolic reprogramming of macrophages to influence their polarisation status, which in turn affects inflammatory responses and skeletal homeostasis [12,60,94]. For example, citric acid has been shown to be a key metabolite in the bone microenvironment of post-menopausal osteoporosis [95], and an MMP-9-specific inhibitor promotes intracellular citric acid synthesis as well as the osteogenic differentiation of bone marrow mesenchymal stem cells (BMSCs), which supports the restoration of bone mass, suggesting that citric acid metabolism plays an important role in bone reconstitution [95]. Notably, it has been shown that citric acid is a key metabolite that affects the bone immune microenvironment by modulating energy metabolic pathways during macrophage polarisation [96]. Experiments using functional scaffolds with controlled citrate ion release capacity showed significant decreases in citric acid levels within bone tissues in an osteoporosis model [96]. The study also revealed that high levels of citric acid accumulate outside M1 macrophages, whereas large amounts are consumed by M2 macrophages [96]. The results suggested that citrate functionalized scaffolds exerted more sensitive inhibitory effects on ATP synthesis and related metabolic enzyme activities (especially on key enzymes of glycolysis, such as phosphofructokinase [PFK], pyruvate kinase, and pyruvate dehydrogenase [PDH]) during polarization of M1 macrophages than during that of M2 macrophages [96]. Mechanistically, citrate blocks the glycolytic process by occupying the active site of PFK, maintaining the metabolic flow of the TCA cycle, thereby contributing to the glycolysis to oxidative phosphorylation shift characteristic of the M2 phenotype, which largely ameliorates bone metabolism in osteoporosis [96]. Therefore, citrate is an important target for controlling macrophage polarisation in osteoporosis, and targeting citrate is expected to provide a new strategy for regulating bone immune metabolism. Although relatively few studies have directly linked citrate to the mechanisms of macrophage polarisation regulation in chronic bone diseases, the properties of citrate as a metabolic intermediate suggest its potential regulatory capacity, and its specific mechanisms of action in various chronic bone diseases need to be further explored.

Fumaric acid is a key component of the tricarboxylic acid cycle and has been extensively investigated in recent years for its pharmacological activity in a variety of diseases. Although studies in the field of bone diseases are still at a relatively early stage, the anti-inflammatory properties of fumaric acid provide a theoretical basis for its application in such an area [97]. In addition, dimethyl fumarate (DMF), a methyl ester of fumaric acid, has excellent anti-inflammatory and anti-oxidant properties and is widely used in the treatment of immuno-inflammatory diseases [98]. DMF modulates M1/M2 macrophage polarisation, thereby reducing inflammation and bone destruction [98]. In a periodontitis model, DMF significantly inhibited osteoclast formation and periodontal tissue destruction, enhanced mitochondrial autophagy, and reduced the infiltration of M1-type macrophages, while increasing the proportion of M2-type macrophages [98]. This mechanism may act through DMF to increase intracellular mitochondrial elongation factor Tu levels and maintain mitochondrial homeostasis [98]. Similarly, treatment of osteoporosis with DMF restored trabecular bone mass in de-ovulated mice due to the activation effect of DMF on Nrf2 [99]. Therefore, DMF is not only able to ameliorate periodontitis by modulating macrophage polarisation but may also play a similar regulatory role in other chronic bone diseases. In the context of OA, monomethyl fumaric acid has shown analgesic and chondroprotective effects by modulating macrophage polarisation [94].

In summary, the mechanism underlying macrophage polarisation by direct regulation of citric acid in most chronic bone diseases still needs to be explored in depth. Although fumaric acid analogues have shown efficacy in several bone disease models, their clinical translation and specific mechanisms in different bone disease microenvironments need to be further verified. Future studies should focus on the systemic role of these metabolites in the bone immune-metabolic network as well as on the development of specific modulators targeting their metabolic pathways, opening new possibilities for the precise treatment of chronic bone diseases [9].

In the complex microenvironment of chronic bone diseases, the tricarboxylic acid (TCA) cycle serves not only as a metabolic hub for energy production but also as a source of intermediate metabolites that act as pivotal signalling molecules in modulating macrophage polarisation. Metabolites such as succinate, itaconate, citrate, and fumarate precisely regulate the phenotypic shift of macrophages by mediating metabolic reprogramming, epigenetic modifications, and specific receptor pathways (e.g., SUCNR1), which ultimately determines the balance between bone resorption and regeneration. However, current research retains significant limitations and contradictions. Firstly, the roles of these metabolites are highly context-dependent; for instance, succinate may exhibit dual effects—promoting both bone destruction and osteogenesis—depending on the pathological stage. Secondly, while itaconate suppresses M1-type inflammation, it may also interfere with the reparative functions of M2 macrophages, leaving the critical threshold for this “immune balance” poorly defined. Prospectively, future studies must move beyond the perspective of isolated metabolites to explore their spatio-temporal distribution and dynamic fluctuations within the osteo-immune network. Elucidating the mechanisms of “cross-cellular communication” of metabolic flux between different bone cells, alongside developing precision targeted delivery strategies for metabolic pathways, will be the key breakthrough in translating fundamental mechanisms into clinical therapies for chronic bone diseases.

## 5. Targeting the ‘Metabolic-Immune’ Axis: Prospects for Innovative Therapies for Chronic Bone Disease

It must be recognised that the application of therapeutic strategies targeting the metabolic–immune axis must be tailored precisely to the specific microenvironment character each distinct chronic bone disease. For instance, interventions for osteoarthritis should focus primarily on resolving synovial inflammation and recalibrating lipid metabolism to arrest cartilage degeneration. Conversely, strategies for osteoporosis must prioritise the restoration of osteoblast–osteoclast homeostasis through modulation of glycolysis and mitochondrial pathways. The targeted therapeutic approaches described below—including nanoparticle delivery systems, extracellular vesicles, biomaterials, and natural products—are designed to meet the precise therapeutic demands imposed by the complex pathological microenvironments of chronic bone diseases.

### 5.1. Nano-Delivery Systems

**Figure 3:**
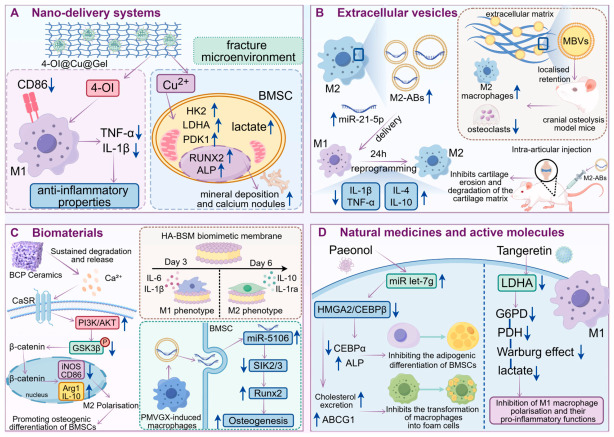
Innovative strategies targeting the “metabolic-immune” axis for chronic bone disorders. This schematic summarises four emerging therapeutic paradigms for restoring bone homeostasis by modulating the immune microenvironment and cellular metabolism. (**A**) Nano-delivery systems: The 4-OI@Cu@Gel nanocomposite hydrogel modulates the fracture microenvironment by suppressing M1 macrophage-mediated inflammation whilst promoting glycolysis (via *HK2*, *LDHA*, and *PDK1*) and osteogenic differentiation in bone marrow mesenchymal stem cells (BMSCs). (**B**) Extracellular vesicles: M2 macrophage-derived apoptotic bodies (M2-ABs) deliver miR-21-5p to reprogramme M1 macrophages into the M2 phenotype, thereby preventing cartilage matrix degradation. Concurrently, matrix-bound nanovesicles (MBVs) enable localised retention to increase M2 macrophages and reduce osteoclastogenesis. (**C**) Biomaterials: Biphasic calcium phosphate (BCP) ceramics release Ca^2+^ to activate the calcium-sensing receptor (CaSR)/phosphoinositide 3-kinase (PI3K)/protein kinase B/β-catenin pathway, driving M2 polarisation and subsequent BMSC osteogenesis. HA-BSM biomimetic membranes chronologically guide M1-to-M2 transitions. Additionally, VGX-1027 microcarrier (PMVGX)-induced macrophages secrete miR-5106, enhancing BMSC osteogenesis via the salt-inducible kinase 2/3 (SIK2/3)-runt-related transcription factor 2 (RUNX2) axis. (**D**) Natural medicines and active molecules: Paeonol upregulates miR let-7g to inhibit both macrophage foam cell formation and BMSC adipogenic differentiation. Tangeretin suppresses M1 polarisation by downregulating key glycolytic enzymes (lactate dehydrogenase A [LDHA], glucose-6-phosphate dehydrogenase [G6PD], pyruvate dehydrogenase [PDH]) and mitigating the Warburg effect. Note: The dark blue upward and downward-pointing arrows indicate the upregulation and downregulation of the corresponding targets, biomarkers, or biological processes, respectively. This figure was generated using Figdraw (www.figdraw.com), with the copyright code IORURe4148.

Nano-delivery systems have emerged as promising intervention strategies for treating chronic bone diseases [100]. This multi-functional and synergistic therapeutic paradigm effectively overcomes the limitations of conventional therapies, whose efficacy is restricted by the short half-life and low bioavailability of drugs. Notably, the targeted modulation of macrophages can be achieved using nanotechnology. For example, Zhao et al. [101] developed a CaCO_3_-mineralised liposome nanoparticle system was able to promote M2 polarisation and inhibit iron death, leading to effective treatment of inflammatory bowel disease, a strategy that may also provide lessons for chronic bone disease. Some researchers have also developed a glucose-functionalized nanoparticle system (berberine-magnetic nanoparticles) that delivers berberine directly to pro-inflammatory M1 macrophages, thereby remodelling the synovial inflammatory microenvironment and effectively slowing OA progression [102]. Similarly, Zha et al. [103] demonstrated that a 4-octyl clathrate itaconate (4-OI)@Cu@Gel nanocomposite hydrogel accelerates fracture healing through metabolic reprogramming of macrophages and bone marrow MSCs (Figure 3). Mechanistically, the 4-OI@Cu@Gel system was able to reduce the expression of the M1-type marker molecule CD86 and the secretion of the inflammatory cytokines TNF-α and IL-1β in macrophages, suggesting that this nano-system can exert anti-inflammatory functions by modulating macrophage polarisation [103]. In addition, compared with the data from the control group, 4-OI@Cu@Gel treatment not only induced an increase in the expression levels of the glycolysis-related genes *HK2*, *LDHA*, and *PDK1* with an increase in lactate production in BMSCs, but also up-regulated the gene expression of the osteogenic markers *RUNX2* and *ALP* and the formation of mineral deposition and calcium nodules, suggesting that 4-OI@Cu@Gel can stimulate glycolysis by promoting the osteogenic differentiation of BMSCs [103]. In vivo experiments also confirmed the effect of 4-OI@Cu@Gel in promoting tissue regeneration and fracture healing in a mouse model of femur fracture [103]. Therefore, this nano-delivery system can improve fracture healing by modulating metabolism and macrophage polarisation; it is expected to be an effective strategy for the treatment of fractures and bone defects. In addition, a biologic nanoparticle engineered to target the gut-bone axis has been developed to effectively ameliorate bone loss associated with inflammatory bowel disease and post-menopause [104]. Structurally, such biologic has a core-shell structure that contributes to the controlled release of butyric acid [104]. Functionally, these nanoparticles are able to remodel the intestinal microbiota, modulate redox balance and macrophage polarisation, restore osteoblast-osteoclast balance—which protects the intestinal barrier and maintains bone homeostasis—, and have significant therapeutic potential for osteoporosis complicated by colitis [104]. Notably, metabolic regulation and macrophage polarisation are not isolated events; there is a profound crosstalk between them. Therefore, nano-systems targeting macrophage energy metabolism can influence the direction of polarisation at the source and thus modulate the bone immune microenvironment. In addition, emerging biomimetic nanotechnologies, such as macrophage membrane-camouflaged nanovesicles, not only inherit the immune escape and chemotaxis abilities of the parent cells but also interact more naturally with the host immune system for safer and more efficient immune modulation [105].

In summary, nano-delivery systems provide unprecedented precision and comprehensiveness in the treatment of chronic bone diseases by integrating multiple functions, such as bone-targeted delivery, metabolic intervention, and immune reprogramming, and represent an important developmental direction in the field of orthopaedic therapy. However, its clinical translation still faces challenges, such as large-scale production, long-term biosafety assessment, and strict regulatory approval.

### 5.2. Extracellular Vesicles

Metabolic reprogramming is closely linked to the functional state of immune cells and is a central intrinsic mechanism that regulates macrophage polarisation. It has been found that EVs of M2-like macrophage origin are able to remodel the metabolic network of osteoclast precursors by delivering specific metabolites, thereby driving their trans-differentiation towards M2-like phenotypes [70]. This finding not only reveals the profound interplay between metabolism and polarisation but also suggests the potential of M2-EVs as a natural and well-targeted nanocarrier for the treatment of bone resorptive diseases. In recent years, the potential of EVs as natural delivery vehicles has been widely recognized. M2 macrophage-derived EVs and MSC-derived EVs have been shown to improve bone healing and formation by promoting M2 macrophage polarisation, providing a new therapeutic avenue for bone repair [70,106,107]. However, whether extracellular vesicles can be loaded with specific metabolic enzymes, ncRNAs, or anti-inflammatory factors for targeted delivery to bone lesions and remodelling of the local bone immuno-metabolic microenvironment remains a question worth exploring.

Matrix-bound nanovesicles (MBVs) are nanoscale vesicles inherent to the extracellular matrix whose lipid bilayer structure protects the contents from degradation and promotes their uptake by target cells. In vivo experiments showed that MBV treatment reduced the number of osteoclasts and TNF-α cells in cranial osteolysis model mice compared to that in controls and also induced the polarisation of their intra-periosteal macrophages towards the M2 phenotype [108]. Therefore, MBV attenuated the inflammatory response induced by UHMWPE particles in cranial osteolysis model mice and effectively improved their immune microenvironments. In addition, near-infrared in vivo imaging showed that locally injected MBVs could form a high concentration enrichment in the cranial lesion region of mice and maintain it for at least 7 days, with a local signal intensity approximately 20 times higher than that of distant organs, such as the liver and kidney [108]. This remarkable local retention ensures that MBVs continue to function at the lesion site, laying the foundation for targeted therapies. Therefore, MBVs exhibit natural anti-inflammatory and anti-bone resorption activities, and their lipid membrane structure and local retention abilities make them ideal platforms for bone-targeted delivery (Figure 3).

Based on a study by Qin et al. [109], M2 macrophage-derived apoptotic vesicles (M2-ABs) and their enriched miR-21-5p provide an innovative strategy for the treatment of chronic bone diseases such as OA. It was shown that M2-ABs were specifically taken up by M1 macrophages, which were reprogrammed to the M2 phenotype within 24 h, significantly elevating the percentage of CD206-positive cells to 79.0% within 48h [109]. In a mouse model of OA generated by anterior cruciate ligamentotomy, articular cavity injection of M2-ABs significantly inhibited cartilage erosion and matrix degradation [109] (Figure 3). Histological analyses further confirmed that the M2-ABs treatment group had a more intact cartilage structure, a significantly higher proportion of M2 macrophages in the synovium, and reduced inflammatory cell infiltration [109]. Mechanistically, M2-ABs regulate macrophage polarisation by delivering miR-21-5p, which inhibits the expression of pro-inflammatory factors (e.g., IL-1β and TNF-α) and promotes the secretion of anti-inflammatory factors (e.g., IL-4 and IL-10) (Figure 3). After inhibiting miR-21-5p function, the M1 to M2 transition induced by M2-ABs is significantly attenuated, suggesting a key role of miR-21-5p in such transition process [109]. In summary, M2-ABs, as natural intercellular communication carriers with the dual functions of targeted immunomodulation and cartilage protection, are expected to develop into apoptotic vesicle-based cell therapies for OA and other chronic bone diseases in the future. In the future, by engineering and modifying specific metabolic enzymes, ncRNAs, or anti-inflammatory factors to be loaded into EVs, we expect to achieve precise and multi-dimensional regulation of the bone immunometabolic microenvironment, thereby providing a new strategy for the treatment of multiple bone immunometabolic diseases.

### 5.3. Biomaterials

The role of biomaterials in the treatment of chronic bone diseases has evolved from a traditional passive scaffolding function to a multi-functional system with active immune regulation [17], one of the core mechanisms of which is to build a microenvironment conducive to bone regeneration by modulating macrophage polarisation status and metabolic reprogramming. In many chronic bone diseases, inflammation persists, leading to an imbalance of M1/M2 polarisation and a vicious cycle of ‘chronic inflammation-bone resorption’ [12]. Biomaterials can overcome this vicious cycle by using various strategies to achieve immunomodulation. Among these strategies, the chemical modification of the surface of the materials is crucial. For example, FPHA, a fluorine-modified biomaterial used for bone regeneration based on immune metabolism, can trigger the metabolic switch of macrophages from glycolysis to oxidative phosphorylation, which can drive the shift to the M2 phenotype and create an immune microenvironment conducive to bone regeneration [50]. Similarly, magnesium ion-doped TiO_2_ nanotube coating (MgN) directs macrophage polarisation towards the M2 anti-inflammatory phenotype through the release of Mg^2+^, which significantly inhibits the expression of pro-inflammatory factors (e.g., IL-6, IL-1β, and TNF-α), while promoting the secretion of anti-inflammatory factors (IL-10 and IL-1ra), VEGF, and bone morphogenetic protein-2 (BMP-2) [110]. Therefore, magnesium-loaded titanium surfaces have good bone immunomodulation and osteogenic ability, providing a new strategy for the surface modification of biomaterial implants.

In addition, in a recent study by Özcolak et al. [111], bone surface topography (BSM) was successfully mimicked by soft lithography, and its effect on the behaviour of RAW 264.7 macrophages was systematically evaluated by combining a chemical modification of collagen type I (Col-I) and hydroxyapatite (HA). The results showed that the immunomodulatory effect of the biomaterial caused cell morphology and cytokine secretion profile alterations. Scanning electron microscopy revealed that, on HA-modified BSM polylactic acid membranes, M1 macrophages exhibited an amoeboid morphology on day 3, which transformed into a spindle-shaped morphology characteristic of the M2 phenotype by day 6, showing a temporal polarisation process [111]. This phenomenon was confirmed by enzyme-linked immunosorbent assay. Namely, in the HA-BSM group, the secretion of the pro-inflammatory factors IL-6 and IL-1β declined after peaking on day 3, whereas the concentrations of the anti-inflammatory factors IL-10 and IL-1ra continued to increase until day 6 [111] (Figure 3). In contrast, neither the topology alone (unmodified BSM) nor a single chemical modification (Col-I modification) induced such pronounced temporal polarisation. Therefore, there is a synergistic effect between the topology of the bionic bone surface and specific bioactive chemical modifications (particularly with HA). This combination of physicochemical properties can effectively mimic the natural bone microenvironment and guide macrophages to complete the temporal polarisation from M1 to M2, thereby providing a key basis for the design of biomaterials with bone immunomodulatory functions. Furthermore, biomaterials, as drug delivery carriers, can achieve the spatiotemporally regulated release of immunomodulatory molecules. Poly(HEMA-co-3APBA) porous scaffolds loaded with luteolin can achieve sustained drug release through borate bonding, effectively induce local M2 polarisation, and promote osteogenic differentiation (by up-regulating the expression levels of osteogenesis-related proteins including RUNX2, ALP, BMP-2, and OPN), which in turn significantly promotes critical-size bone defect (csbd) repair [112]. More importantly, metabolic regulation has emerged as a new hub that connects biomaterials to macrophage function.

In a recent study, a bone immunomodulatory biomaterial, PMVGX, was designed. PMVGX can reprogram macrophage mitochondrial metabolism, induce macrophage polarisation towards the M2 phenotype, and enhance cytosolic burial, thereby improving the bone immune microenvironment and promoting bone repair [76]. Additionally, PMVGX induces macrophages to release miR-5106-containing exosomes, which act on BMSCs via paracrine secretion to promote osteogenic differentiation and further accelerate bone regeneration via the salt-inducible kinase 2/3-RUNX2 pathway [76] (Figure 3). Similarly, fiber scaffolds synergistically ameliorate impaired energy metabolism, chronic inflammation, and vascular insufficiency by regulating endogenous VEGF recruitment and immune factor secretion, ultimately restoring the regenerative potential of the aging bone [113]. In vivo experiments confirmed that the scaffold significantly enhanced bone defect repair and angiogenesis in both aged mice and OVX rats [113]. The study provided a paradigm shift from ‘single-factor delivery’ to ‘endogenous ecological site engineering’ for bone regeneration in the elderly, which has potential for clinical translation. Another study by Zhang et al. [114] found that biphasic calcium phosphate ceramics continuously release Ca^2+^ during degradation, and these ions can act as key metabolic signalling molecules after uptake by macrophages, initiating the downstream Wnt/β-catenin signalling pathway through activation of calcium-sensitive receptors (CaSRs) on the cell membrane. This Ca^2+^-CaSR-Wnt/β-catenin axis constitutes a specific pattern of metabolic regulation that translates extracellular inorganic ion signals into intracellular metabolic and transcriptional reprogramming events that convert macrophages towards a pro-repair M2 phenotype and promote the differentiation of MSCs into osteoblasts, ultimately leading to osteoinduction [114] (Figure 3). Therefore, the study revealed the mechanism by which biomaterials affect immune cells by modulating the ionic metabolic environment, providing an important basis for the design of immunomodulatory biomaterials.

In summary, modern biomaterials are no longer limited to providing mechanical support but act as ‘immune engineers’ to dynamically reshape the local immune ecology by integrating the intrinsic properties of materials, controlled release systems, and metabolic interventions. Future development directions should focus on the construction of a closed-loop system with the ability of feedback regulation, which should be combined with the patient’s individual immune characteristics for precise medical design, to promote the treatment of chronic bone diseases from ‘replacement and repair’ to the ‘functional regeneration’ of the new stage.

### 5.4. Natural Medicines (Herbal Monomers) and Active Molecules

The mechanism of action of natural drugs and active molecules in the regulation of chronic bone diseases is increasingly understood as a multi-dimensional regulatory process through the ‘metabolic-immune’ axis [115,116,117]. The bioactive molecule naringenin, a flavonoid glycoside, has been shown to have therapeutic potential for the alleviation of inflammatory arthritis and osteoporosis [118]. The findings revealed that naringenin significantly inhibits LPS-induced macrophage differentiation towards a pro-inflammatory M1 phenotype and promotes a shift towards an anti-inflammatory M2 phenotype. Naringenin also inhibits the expression of pro-inflammatory cytokines (e.g., iNOS, TNF, IL-1β, and IL-6) and up-regulates immune-suppressing mediators (e.g., IL-10, Arg-1) [118]. Furthermore, naringenin significantly inhibits osteoclast differentiation, which is associated with the inhibition of the NF-κB and MAPK signalling pathways [118]. These results strongly confirm the potential of bioactive molecules in alleviating the development of chronic bone diseases by modulating immune function. In addition, herbal compounds may attenuate chronic bone disease immune responses by acting on key inflammatory signalling pathways. For example, Bushen Quhan Zhiwang decoction was found to effectively down-regulate osteoblast cytokines and pro-inflammatory cytokines (down-regulating the expression of receptor activator of nuclear factor kappa-B ligand, TNF-α, IL-1β, and IL-6), which inhibits osteoclastogenesis and bone resorption function, and was found to have osteoprotective effects on rats with collagen-induced arthritis [119]. This specific mechanism may be related to the inhibition of the TRAF6/p38/JNK MAPK pathway by Bushen Quhan Zhiwang decoction [119]. The effects of natural products present a systematic and multi-level nature. On the one hand, they can directly target osteoblasts; on the other hand, they affect bone metabolism through an indirect pathway, that is, by regulating the function of immune cells. As a key hub connecting immunity and bone reconstruction, the polarisation of macrophages from pro-inflammatory M1-type to anti-inflammatory M2-type is crucial for initiating bone regeneration.

Further, a study investigated the mechanism of action of the active substance Paeonol in the traditional Chinese medicine danpi in an atherosclerosis-osteoporosis co-morbidity model. It was confirmed that Paeonol regulates lipid metabolism and macrophage behaviour by upregulating miR let-7g expression and inhibiting the downstream HMGA2/CEBPβ signalling pathway [120] (Figure 3). In vivo experiments revealed that Paeonol not only reduced atherosclerotic plaques and lipid accumulation but also lowered bone loss and bone marrow lipogenesis [120]. In vitro experiments have shown that Paeonol treatment inhibits macrophage-to-foam cell conversion, promotes cholesterol efflux, and reduces esterification rates [120]. Paeonol improves bone metabolic homeostasis by inhibiting the lipidogenic differentiation of BMSCs, reflecting its multi-target regulatory properties [120]. Therefore, Paeonol provides a molecular basis for atherosclerosis-osteoporosis combination therapy through miR let-7g-mediated metabolic and macrophage regulation, highlighting its potential as a pleiotropic active molecule. Another study found that Tangeretin, a naturally occurring polymethoxyflavonoid delivered via a self-nanoemulsifying drug delivery system, significantly improves rheumatoid arthritis (RA) symptoms [121]. Tangeretin regulates energy metabolism by downregulating glycolysis—which manifests as a recovery (increase) in basal respiration and maximum respiration that had been suppressed by M1 polarisation—whilst also downregulating lactate secretion. This is mainly achieved by inhibiting key enzymes, such as LDHA, glucose-6-phosphate dehydrogenase, and PDH [121] (Figure 3). Therefore, Tangeretin inhibits M1 macrophage polarisation and function by modulating metabolic reprogramming, providing a promising approach for effective relief of rheumatoid arthritis symptoms.

In summary, natural drug monomers and active molecules do not simply inhibit inflammation or promote osteogenesis but rather act through a complex metabolism-immunity-osteogenesis network that is finely tuned. They may act in multiple ways simultaneously, inhibiting the activation and metabolic reprogramming of pro-inflammatory immune cells, promoting the differentiation of anti-inflammatory immune cells, regulating key signalling pathways, and ultimately balancing the activities of OCs and OBs to achieve a fundamental intervention in chronic bone diseases. Future studies should combine the cutting-edge technologies of metabolomics, immunomics, and network pharmacology to further analyse specific molecular targets and pathways of action and provide a scientific basis for the development of safer and more effective therapeutic strategies for bone diseases.

## 6. Conclusions

**Figure 4:**
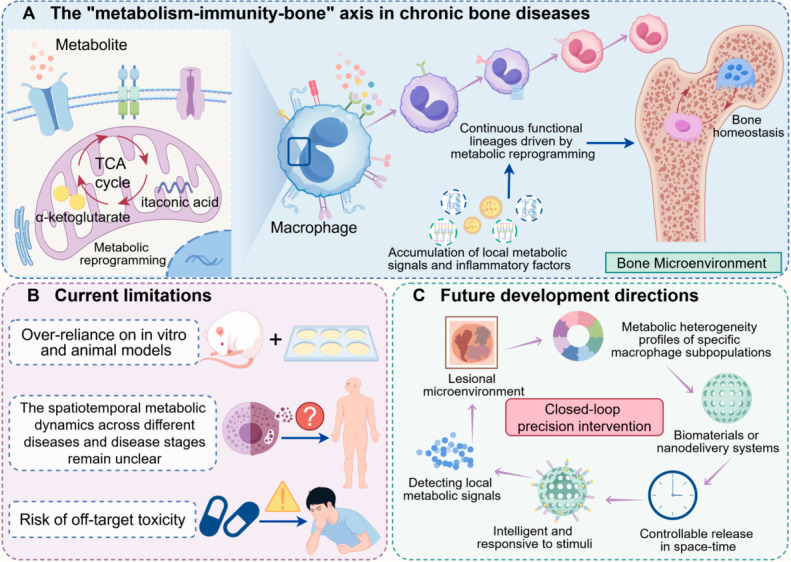
Overview of the ‘metabolic-immune-bone’ axis in chronic bone diseases, current limitations and future prospects. (**A**) The ‘metabolic-immune-bone’ axis. Macrophage polarisation is not a simple binary switch, but rather a continuous functional spectrum driven by metabolic reprogramming. Key metabolic intermediates in the tricarboxylic acid cycle (TCA cycle) (such as α-ketoglutarate and itaconic acid) tightly couple cellular metabolic states with gene expression. The accumulation of local metabolic signals and inflammatory factors determines the functional spectrum of macrophages, thereby regulating bone homeostasis within the bone microenvironment. (**B**) Current limitations of translational research. Current understanding is constrained by an over-reliance on in vitro and animal models, which cannot fully replicate the complexity of human skeletal pathophysiology. Furthermore, the spatiotemporal metabolic dynamics of specific macrophage subpopulations across different diseases and disease stages remain unclear. Systemic metabolic interventions also carry significant risks of off-target toxicity. (**C**) Future directions. Future therapeutic strategies will shift towards precision interventions. This includes mapping the metabolic heterogeneity of specific macrophage subpopulations within the tumour microenvironment. Furthermore, emphasis will be placed on developing smart, stimulus-responsive biomaterials or nanodelivery systems capable of detecting local metabolic signals, to enable the spatiotemporal controlled release of immunomodulatory substances. This figure was generated using Figdraw (www.figdraw.com), with the copyright code APATA88108.

The core pathological mechanism underlying chronic bone diseases lies in the mutual interplay between an imbalance in macrophage polarisation and dysregulated metabolic reprogramming. However, macrophage polarisation is not a simple M1/M2 binary switch, but rather a continuous functional spectrum driven by metabolic reprogramming, the heterogeneity of which is determined by factors such as signal-specific transcriptional programmes, epigenetic modifications, and the timing of signal exposure. Among these, metabolic intermediates such as α-ketoglutarate, itaconic acid and citrate couple metabolic state with gene expression through epigenetic modifications (such as Jmjd3-mediated H3K27me3 demethylation), thereby providing a mechanistic explanation at the level of gene regulation for metabolic reprogramming. It is particularly noteworthy that the functional state of mitochondria not only provides energy support but also profoundly influences macrophage fate determination through mechanisms such as phagocytosis, reactive oxygen species regulation and mitochondrial transfer. Based on these findings, this paper proposes the ‘metabolism-immunity-bone’ axis as a key theoretical framework for understanding the pathogenesis of bone diseases (Figure 4), emphasising that metabolic reprogramming serves as the central hub linking immune regulation and bone homeostasis. Consequently, precise spatiotemporal interventions targeting specific metabolites may emerge as a highly promising therapeutic strategy for regulating the onset and progression of various bone diseases.

Nevertheless, current understanding remains limited (Figure 4). Firstly, an over-reliance on in vitro and animal models restricts the reliability of translational research. Most of the metabolic regulatory mechanisms discussed in this paper are derived from mouse models; however, the pathophysiological environment of human bone exhibits unique metabolic and immunological complexities that are difficult to accurately replicate in rodents. Secondly, the heterogeneity of macrophages implies that different subpopulations may possess distinct metabolic characteristics; current research has not yet fully elucidated the spatiotemporal metabolic dynamics of specific subpopulations across different diseases and disease stages. Finally, the systemic application of metabolic interventions carries a significant risk of off-target toxicity. Modulating ubiquitous metabolic pathways (such as glycolysis or the citric acid cycle) requires highly precise, localised delivery mechanisms to avoid disrupting systemic metabolic homeostasis.

Furthermore, we speculate that future management of chronic bone diseases will shift from non-specific anti-inflammatory strategies towards precise metabolic regulation, which represents a promising direction for development. Achieving this goal will require the integration of cutting-edge technologies, such as single-cell spatial transcriptomics, to map the metabolic heterogeneity of specific macrophage subpopulations within the pathological microenvironment (Figure 4). Concurrently, there is a need to develop intelligent, stimulus-responsive biomaterials and nanodelivery systems capable of sensing local metabolic signals, thereby enabling the spatiotemporally controlled release of immunomodulatory payloads (Figure 4). By integrating these interdisciplinary insights, future research is expected to transform descriptive pathophysiological observations into predictive and personalised therapeutic strategies, thereby providing stage-specific intervention protocols for various chronic bone diseases. In summary, although the existing literature has amply demonstrated that metabolic reprogramming fundamentally regulates the functional plasticity of macrophages, most studies remain confined to single-disease paradigms and are constrained by the traditional M1/M2 classification, resulting in a significant lack of in-depth elucidation of spatiotemporal immune-metabolic dynamics across different stages of disease progression. Consequently, as an advanced theoretical framework, the ‘metabolic-immune-bone’ axis requires further refinement supported by additional cross-disease, multi-stage research.

## Data Availability

No new data were created or analyzed in this study. Data sharing is not applicable to this article.

## Abbreviations

The following abbreviations are used in this manuscript:

MSCs	Mesenchymal stem cells
NF-κB	Nuclear factor kappa B
GMSCs	Gingival tissue-derived MSCs
TNF-α	Tumour necrosis factor-α
OVX	Ovariectomised
RKIP	Raf kinase inhibitory protein
scRNA-seq	Single-cell RNA sequencing
IL-4	Interleukin-4
TLR	Toll-like receptor
LPS	Lipopolysaccharide
AMPK	AMP-activated protein kinase
GLP-1R	Glucagon-like peptide-1 receptor
IMP2	Insulin-like growth factor 2 mRNA-binding protein 2
TGF-β	Transforming growth factor-β
IL-10	Interleukin-10
FSH	Gonadotropins
NAD^+^	Nicotinamide adenine dinucleotide
MAS	Malate-aspartate shuttle
CREB	cAMP response element-binding protein
T2DOP	Type 2 diabetic osteoporosis
FPHA	Fluorinated porcine hydroxyapatite
iNOS	Inducible nitric oxide synthase
OA	Osteoarthritis
IL-1β	Interleukin-1β
LDHA	Lactate dehydrogenase A
AGEs	Advanced glycosylation end products
HFD	High-fat diet
eWAT	Epithelial white adipose tissue
OPN	Osteopontin
KEGG	Kyoto Encyclopaedia of Genes and Genomes
ACOD1	Aconitic acid decarboxylase 1
TCA cycle	Tricarboxylic acid cycle
M2-EVs	M2-type macrophage-derived extracellular vesicles
OCPs	Osteoclast precursors
αKG	Alpha-ketoglutarate
H3K27me3	Histone 3 lysine 27 trimethylation
PMVGX	Microcarrier loaded with VGX-1027
VEGF	Vascular endothelial growth factor
RUNX2	Runt-related transcription factor 2
SUCNR1	Succinate receptor 1
IL-6	Interleukin-6
IFN-β	Interferon-beta
OI	4-octyl clathrate
SAON	Steroid-associated osteonecrosis
BMSCs	Bone marrow mesenchymal stem cells
PFK	Phosphofructokinase
PDH	Pyruvate dehydrogenase
DMF	Dimethyl fumarate
4-OI	4-octyl clathrate itaconate
MBVs	Matrix-bound nanovesicles
M2-ABs	M2 macrophage-derived apoptotic vesicles
BMP-2	Bone morphogenetic protein-2
BSM	Bone surface topography
Col-I	Collagen type I
HA	Hydroxyapatite
CaSRs	Calcium-sensitive receptors
